# Hypoxic 3D in vitro culture models reveal distinct resistance processes to TKIs in renal cancer cells

**DOI:** 10.1186/s13578-017-0197-8

**Published:** 2017-12-16

**Authors:** Zofia F. Bielecka, Agata Malinowska, Klaudia K. Brodaczewska, Aleksandra Klemba, Claudine Kieda, Paweł Krasowski, Elżbieta Grzesiuk, Jan Piwowarski, Anna M. Czarnecka, Cezary Szczylik

**Affiliations:** 1 Department of Oncology with Laboratory of Molecular Oncology, Military Institute of Medicine, Szaserów 128, 04-141 Warsaw, Poland; 2 School of Molecular Medicine, Warsaw Medical University, Księcia Trojdena 2a, 02-091 Warsaw, Poland; 3 Environmental Laboratory of Mass Spectrometry, Polish Academy of Sciences, Institute of Biochemistry and Biophysics, Pawińskiego 5a, 02-106 Warsaw, Poland; 4 Centre for Molecular Biophysics, Cell Recognition and Glycobiology, UPR4301-CNRS, rue Charles Sadron, 45071 Orléans, France; 5 Department of Molecular Biology, Polish Academy of Sciences, Institute of Biochemistry and Biophysics, Pawińskiego 5a, 02-106 Warsaw, Poland

**Keywords:** Hypoxia, Tyrosine kinase inhibitors, Drug resistance, 3D cell culture, Renal cell carcinoma, Papillary, Clear-cell

## Abstract

**Background:**

The aim of this study is to determine the effect of hypoxia on axitinib and sorafenib-treated renal cell carcinoma (RCC) cells. Hypoxia is a crucial factor influencing transcription process via protein modulation, which was shown i.e. in pancreatic cancer. Until now, hypoxia has been defined as associated with poorer outcome and inducing chemotherapy resistance in solid tumors. The unique phenomenon of pseudo-hypoxia connected with *vhl* mutation was observed in clear-cell, but not in papillary RCC, and the treatment of this subtype of cancer is still challenging. Despite the introduction of new antiangiogenic targeted therapies (*inter alia* tyrosine kinase inhibitors, TKIs), patients still develop both primary and acquired resistance. Overcoming resistance to TKIs, also in papillary RCC, may be possible by finding significantly modified protein expression. To do this, hypoxic 3D in vitro models must be developed to mimic both molecular pathways typical for low oxygen tension and cell–cell dynamics in tumor-like spatial structures.

**Results:**

Clear-cell and papillary renal cell carcinoma (cc and pRCC) cell lines were used in the study to determine the impact of hypoxia on primary drug resistance phenomenon previously observed in papillary, but not in ccRCC. Resistance was confirmed in monolayer culture and in 3D models in soft agar and suspension culture. Human papillary kidney cancer stem-like cells (HKCSCs) cultured in hypoxia developed resistance to sorafenib, while when cultured in normoxia resistance to axitinib has developed. Flow cytometry revealed that hypoxia decreased proliferation rates in all investigated RCC cells. In HKCSCs, there was an increase of quiescent cells (Ki67−) and percentage of cells arrested in S phase. It also appeared that map2k1 and eif4b protein expression is altered in papillary RCC resistant to tested drugs at different oxygen tensions. Also, HKCSCs did not express *vegfr*-*1, braf nor c*-*kit*, TKIs target receptors, which were present in ccRCC cells sensitive to TKI treatment.

**Conclusions:**

The results confirm that low oxygen tension affects RCC cells. Hypoxia facilitates induction of sorafenib resistance in pRCC and induces map2k1 overexpression, while normoxic axitinib-resistant cells up-regulated eif4b. Further studies may determine if map2k1 or eif4b proteins play a role in pRCC resistance to TKIs. It is also of interest to establish if other than *vegfr*-*1, braf, c*-*kit* receptors can serve as potential molecular targets for more effective anti-RCC strategies.

## Background

Renal cancers accounted for 3–5% of all malignancies in the adult population of the United States in 2013; 90–95% of kidney neoplasms are renal cell carcinomas (RCCs). Although initially RCC was believed to represent a uniform malignant phenotype, it is now known that it constitutes a diverse group of cancers arising from the kidney tubule [1]. Clear cell (ccRCC), papillary (pRCC) and chromophobe (chRCC) reflect the main histological subtypes of RCC [1–3]. RCC subtypes are defined by specific genetic abnormalities including von Hippel Lindau (*vhl*), hepatocyte growth factor receptor (*c*-*met*), Birt-Hogg-Dubé (*bhd*) and fumarate hydratase (*fh*) gene mutations, which was confirmed in both classical genetics and in genomic studies [2, 4, 5]. Proliferative activity of all RCC cells, regardless of its subtype, is dependent on constitutive activation of receptor tyrosine kinases (RTKs). These RTKs are targeted primarily by tyrosine kinase inhibitors (TKIs)—sorafenib, sunitinib, pazopanib, axitinib or cabozantinib [6, 7]. Response to targeted therapies varies in its effectiveness and among RCC subtypes [8]. For the purposes of this study, several TKIs widely used in RCC treatment according to newest ESMO guidelines [9], were chosen: sunitinib, which binds 73 kinases in addition to its main target, vegfr-2; sorafenib, which binds 40 additional kinases; and axitinib, which is the most selective, with a limited number of targets, mostly vegfr [10].

Sunitinib is primarily a potent inhibitor of vegfr-1, vegfr-2 (vascular endothelial growth factor receptors 1 and 2), flt-3 (FMS-related tyrosine kinase 3), c-kit (also called cd117—stem cell factor receptor/proto-oncogene *c*-*kit*), pdgfrα and pdgfrβ (platelet-derived growth factor receptors). It inhibits the growth of cancer cell lines stimulated by *vegf, scf* (skp, cullin, f-box containing complex), or *pdgf* and induces apoptosis [11, 12].

Like sunitinib, sorafenib inhibits activity of vegfr2 and vegfr3 but not vegfr-1, flt-3, c-kit and pdgfr [13]. It has been shown that sorafenib decreases tumor cell proliferation via *raf*-*1* (as well as wild-type *braf* and *v599e braf*) inhibition and that it effectively blocks the *raf/mek/erk* signaling pathway in cancer cells [14]. It is also clear that sorafenib inhibits cancer cell proliferation in a dose-dependent manner and induces cancer cell apoptosis as previously shown in the hepatocellular carcinoma (HCC) or leukemia models [14, 15]. Sorafenib was further shown to induce endothelial cells’ apoptosis in tumors. Thus, a dual inhibitor of *raf* kinase and vegfrs targets both cancer cells proliferation and cancer angiogenesis [13, 16].

Axitinib is a more selective second-generation inhibitor of vegf-1, 2, and 3, pdgfrβ and c-kit [10]. Although axitinib is mostly known as an inhibitor of endothelial cell survival and new tube formation as well as an inhibitor of protein kinase B (*pkb*, *akt*), nitric oxide (NO), and extracellular signal-regulated kinase (*erk*) signaling in endothelial cells [17]. It has also been shown that axitinib exerts direct cytotoxic activity against cancer cells. In cancer cells, axitinib inhibits cell growth and viability in a dose-dependent manner, causing a G_2_/M cell-cycle arrest [18, 19]. Axitinib also blocks *wnt/β*-*catenin* signaling in cancer cells [20]. It was also shown to inhibit proliferation of patient-derived glioblastoma cancer stem cells [21] and was used in potential myxoma virus-based treatment directed against brain tumor-initiating cells [22].

Although these tyrosine kinase inhibitors have been applied to clinical settings, and their usability is still developing, the underlying molecular mechanisms behind anti-tumor activity remain unclear. Precise knowledge of up- and downregulated proteins in TKI-treated cells as well as of TKIs’ in vitro effect in normoxia and hypoxia may help maximize treatment efficacy.

Until now, hypoxia has been defined as associated with poorer outcome and inducing chemotherapy resistance when present in solid tumors. Moreover, it has been shown that hypoxia does not necessarily act via hypoxia inducible factors—associated pathways [23]. Interestingly, hypoxia but not normoxia was shown to modulate transcription process via protein upregulation in pancreatic cancer [24]. Therefore, our primary aim in this research was to evaluate the effect of hypoxia on TKI-treated renal cancer cells of various histopathological origin, including papillary RCC.

Today, research in drug resistance research occurs in the most rapidly evolving areas of solid tumor molecular oncology research [25], however, the impact of hypoxia on renal cancer cells with primary resistant cell subpopulations has not been fully characterized in any RCC cell culture bio-mimic model until today, also not in a 3D cell culture hypoxic model [26]. 3D cell culture models better mimic in vivo conditions [27]. Moreover, cell growth dimensionality is strictly related to oxygen status. Pathologically relevant cell culture models in proper oxygen tension are required to study the complex physical and chemical processes by which the cancer microenvironment mediates drug resistance [25]. Understanding these processes is especially significant because the hypoxia signaling pathway is frequently de-regulated in clear-cell renal cell carcinoma due to *vhl* mutations [28–30] and limited information is available on intratumoral hypoxia-mediated signaling abnormalities in pRCC or ccRCC. In most hypoxia signaling studies, nephrectomy samples are analyzed [31] and only hypoxia inducible factors (hifs) mRNA levels are investigated [32], but no functional data is available. Hypoxia must be further investigated to explain efficacy of TKI treatment in RCC.

Therefore, the secondary aim of this study is to analyze TKI response in low oxygen tension in clear-cell and non-clear cell RCCs and in human kidney cancer stem cells.

Until today, sorafenib has been recognized as more effective in oxygenated (normoxic) environments [33]. In adherent monolayer 2D cell cultures, sorafenib inhibits viability of 786-O ccRCC cells under normoxia more effectively than in hypoxia. In 2D cell culture, hypoxia promotes 786-O cells resistance to sorafenib and its invasiveness. Hif-2α and cox-2 signaling pathways were shown to be responsible for this phenomenon [34]. Moreover, the ACHN cell line was shown to be generally sensitive to hypoxia, characterized by slower proliferation and subsequent resistance to sorafenib (anti-cancer, anti-proliferative activity) [33]. At the same time, Caki-1 and 786-O cells expressing wild type *vhl*—compared to Caki-2 and 786-O mutant *vhl* cells—were shown to be twofold more resistant to the anti-proliferative effects of sorafenib under hypoxic conditions. No difference in resistance was observed under normoxic conditions [35]. Taking this into account, our tertiary goal is to compare TKI response in hypoxic and normoxic conditions in a 3D culture model.

All steps of the study—(1) RCC cells TKI response, (2) human papillary kidney cancer cells (HKCSCs) drug resistance, and (3) TKI response in different oxygen tension—represent one spectrum of co-dependent phenomena. The ultimate aim of this study is to explain how hypoxia affects TKIs direct impact on renal cancer cells and how low oxygen tension and cancer cell–cell interactions in 3D structures modulate RCC tyrosine kinase inhibitors’ resistance. Understanding the hypoxia-induced RCC TKI-resistant phenotype is crucial to develop novel in vitro study models for testing targeted therapies.

**Fig. 1:**
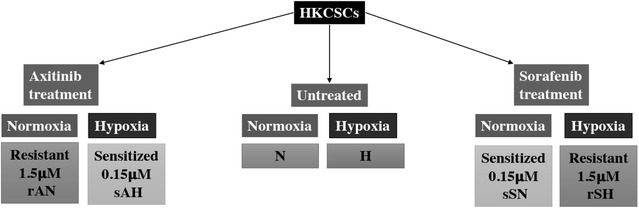
Schematic presentation of the compared treatment and oxygen groups of HKCSCs

Throughout the article, we use several abbreviations for human papillary renal cancer cells (HKCSCs), which during the study: (1) developed resistance to axitinib in normoxia—those are marked *rAN*; (2) developed resistance to sorafenib in hypoxia—those are marked *rSH*; (3) remained sensitive to axitinib in hypoxia (i.e. were sensitized)—marked *sAH*; (4) remained sensitive (i.e. sensitized) to sorafenib in normoxia—marked *sSN*, (5) were untreated in normoxia—*N*, and (6) were untreated in hypoxia—*H*. These abbreviations comprise only HKCSCs’ cell line, since none of other cell lines used in the study developed TKI-mediated resistance and thus were not further investigated. Cells were compared either in one oxygen tension group, i.e. only normoxic vs only hypoxic samples, or in one treatment group regardless of oxygen tension, i.e. axitinib resistant and sensitized vs untreated, etc. The mechanism of cells comparison is presented on Fig. 1.

## Methods

### Cell lines

Each cell line was obtained directly from a cell bank that performs individual cell line characterization. After delivery, cells were sub-cultured after the first passage for several stocks, which have been used in further years. The stocks used in this research were independently de-frozen in 2014–2015 and used for no more than 6 months.

HKCSCs have been originally obtained from a single donor diagnosed with papillary RCC; the cell line was obtained from Celprogen Inc. (Cat. No. 36117-44) and primarily cultured in monolayer in a medium recommended by the producer: Human kidney cancer stem cell complete medium with serum and antibiotics (Celprogen, Inc., Cat. No. M36117-44S) and passaged using a trypsin/EDTA solution (Sigma-Aldrich). HKCSCs have been previously described by Celprogen, Inc. and are positive for *pax2, cd44, cd133, ssea ¾, c*-*met, oct3/4, aldh* and *telomerase.* HKCSCs form tumors in SCID mice and is *vhl*-proficient (data not shown).

The cell lines described below were obtained from ATCC. Caki-1, human metastasis of clear-cell renal cell carcinoma to the skin, expresses wild-type *vhl* (*vhl*-proficient) and forms tumors in immunocompromised mice (ATCC^®^ HTB-46™), and 769-P, human primary clear-cell renal cell carcinoma with *vhl* mutation (*vhl*-deficient), which forms tumors in NOD/SCID mice (ATCC^®^ CRL-1933™), were cultured primarily in monolayer in RPMI 1640 supplemented with GlutaMAX™-I (Gibco, Cat. No. 61870036) as well as 10% FBS (PAN Biotech, Cat. No. P40-1301) and 1% 100 U/ml penicillin/streptomycin (Life Technologies, Cat. No. 15140122). All cells were seeded in T75 flasks and passaged every 2–3 days. Cells were cultured either in normoxia, in a humidified, 5% CO_2_- and 20% O_2_-containing atmosphere or in hypoxic conditions, which were generated in a humidified hypoxic incubator with 1% O_2_, 5% CO_2_ and balanced N_2_ content.

Cells were visualized using an inverted Olympus microscope with Olympus camera UC30 (Serial No. 14310982) and Olympus Entry Cell Sense 1.8.1 software (Serial No. PY8HDQECP6Q, core version XV 3.8.).

### Tyrosine kinase inhibitors dose response

Clinically relevant maximum doses of sunitinib, sorafenib and axitinib were calculated using their peak concentrations in serum or plasma. Sunitinib was obtained from Sigma-Aldrich (Cat. No. PZ0012). Axitinib was also obtained from Sigma Aldrich (Cat. No. PZ0193), and sorafenib was obtained from Cayman, Inc. (Cat. No. BAY-43-9006). TKIs were first dissolved in DMSO (%DMSO < 2% in the final concentration in used media). For HKCSCs, 769-P and Caki-1 monolayer cell cultures, following drug concentrations were used in normoxia and in hypoxia for a primary screening of their response to drug treatment: sunitinib and sorafenib—C_1_ = 0.15 µM (maximum clinically relevant dose administered to patients) [36, 37], C_2_ = 1.5 µM, C_3_ = 15 µM, C_4_ = 150 µM; axitinib—C_1_ = 0.07 µM (maximum clinically relevant dose administered to patients) [38], C_2_ = 0.15 µM, C_3_ = 1.5 µM, C_4_ = 15 µM. Subsequent choices of doses used for further research resulted from an AlamarBlue^®^ cell lines viability assessment. For resistant HKCSCs cell lines, doses were 10× higher than for sensitized HKCSCs cells. For soft agar colony formation assay, HKCSCs were treated with sorafenib and axitinib in concentrations of 0.15 and 1.5 µM.

769-P and Caki-1 cell lines that did not develop resistance were subjected to treatment only with 0.15 µM of both TKIs. Subsequent experiments excluded 769-P and Caki-1 as non-resistant cell lines.

### AlamarBlue^®^ viability assay and growth curves

Cells were seeded on flat-bottom, 96-well plates (Thermo Fisher Scientific, Cat. No. 07-200-90) in the following concentrations to achieve their logarithmic phase of growth: HKCSCs—1000 cells/well, 769-P—1500 cells/well, Caki-1—2000 cells/well. Each condition was repeated four times. AlamarBlue^®^ was performed in monolayer culture conditions. After 24 h, the medium was changed for the appropriate medium containing TKI. HKCSCs, 769-P and Caki-1 cell lines were cultured in normoxia and hypoxia in the following drug concentrations: sorafenib—C_1_, C_2_, C_3_, C_4_, axitinib—C_1_, C_2_, C_3_, C_4_. AlamarBlue^®^ viability assay was performed according to proper protocol (Life Technologies™, Cat. No. DAL1025). Ten microlitre AlamarBlue^®^ was added to the 90 µl medium with cells to reach a final concentration of 10% in each well, as previously described in the literature [39]. Cells cultured in medium without treatment were used as controls. Growth curves for HKCSCs, 769-P and Caki-1 cell lines in normoxia and hypoxia were obtained by counting the number of cells per 1 ml each day during 6 days of monolayer cell culture in T25 flasks using an MOXI™ Z Mini Automated Cell Counter Kit (ORFLO Technologies, Cat. No. MXZ001). Percentage of Alamar blue reagent’s reduction showed the percentage of cells that reduced resazurin to resorufin, which were also metabolically active and thus alive.

### Soft agar colony formation assay

Colony formation assay was performed on HKCSCs, 769-P and Caki-1per the STEMTag 96-well Stem Cell Colony Formation Assay (Cell Biolabs, Inc., Cat. No. CBA-325). Each sample as well as control was prepared in triplicate. Pictures of colonies were taken on day 17 (data not shown) and day 30 under an inverted microscope. The medium was changed every 4–5 days. Cells were cultured in normoxia (pO_2_ 18.75%) and hypoxia conditions (pO_2_ 1%, SANYO MCO-5 M incubator).

### Suspension cell cultures

Both clear-cell and papillary RCC were studied using two in vitro models (adherent cell culture and colony formation assay). After noticing the primary resistance to TKI-like phenomenon in papillary RCC cells, the results were confirmed in a third in vitro model—suspension culture—using only HKCSCs, which were either untreated in normoxia (*N*) or hypoxia (*H*), sensitized to sorafenib in normoxia (*sSN*) and to axitinib in hypoxia (*sAH*) or resistant to sorafenib in hypoxia (*rSH*) and to axitinib in normoxia (*rAN*).

HKCSCs were seeded in T25 polystyrene flasks (Corning^®^, VWR Cat. No. 29186-010) and cultured in StemXVivo Mesenchymal Stem Cell Suspension Medium containing EMT (epithelial to mesenchymal transition) Supplement (R&D Systems, Cat. No. CCM004), which allows for the creation of 3D structures in a suspension culture. Cells were cultured as follows:

1. In normoxia: (a) in 1.5 µM axitinib (cells resistant to axitinib in normoxia: *rAN*), (b) in 0.15 µM sorafenib (cells sensitized to sorafenib in normoxia: *sSN*), (c) untreated (normoxia control: *N*);
2. In hypoxia: (a) in 1.5 µM sorafenib (cells resistant to sorafenib in hypoxia: *rSH*), (b) in 0.15 µM axitinib (cells sensitized to axitinib in hypoxia: *sAH*), (c) untreated (hypoxia control: *H*).

TKIs were added to the cell culture on day 3 so that treatment would impact formed 3D structures. On day 6, total protein was isolated.

### Muse™ cell cycle and proliferation analysis

To test whether cell cycle is affected in HKCSCs, depending on oxygen concentration and/or TKI addition, untreated, sensitized and resistant cells were examined both in normoxia and hypoxia. HKCSCs cells were seeded in T25 polystyrene flasks as described in “Soft agar colony formation assay” of this report. On day 6, cells were prepared for Muse™ Cell Analyzer dedicated tests: Muse™ Cell Cycle Assay Kit (Merck Millipore, Cat. No. MCH100106) and Ki67 Proliferation Assay Kit (Merck Millipore, Cat. No. MCH100114) according to the manufacturer’s instructions. Collected cell aggregates were disrupted using warm accutase prior to permeabilization and fixation. An average of 5000 cells was analyzed for each condition. Fractions of cells in G0/G1, S and G2/M phase as well as fractions of proliferating cells were determined on day 6 of cell culture.

### Protein isolation and mass spectrometry

Mass spectrometry and western blot analysis were both performed on untreated and resistant groups of papillary RCC cells (*rAN, N, rSH, H*). Briefly, total protein was isolated using an RIPA buffer (Sigma-Aldrich, Cat. No. R0278) and a Protease Inhibitor Cocktail (Sigma-Aldrich, Cat. No. P8340). Protein pellet was obtained and densed after overnight incubation under acetone for mass spectrometry analysis. Three biological replicates were prepared for each analytical group. Protein extracts were trypsin digested overnight with 10 ng/µl trypsin, then reduced with 5 mM TCEP for 60 min at 60 °C; cystein thiol residues were blocked with 10 mM MMTS for 10 min at room temperature. Resulting peptide mixtures were analyzed with a LC–MS system composed of a nanoHPLC chromatograph (nanoAcquity, Waters), directly coupled to the ion source of the LTQ Orbitrap Velos working in MS mode (profile datasets, no data sequencing). Tandem mass spectrometry for peptide sequencing was carried out separately for each group by the following procedure: peptide mixtures from all biological replicates were mixed into a single sample and measured in triplicate. To increase the proteome coverage, each of the three MS/MS measurements covered different m/z ranges: 300–600, 600–900 or 900–2000 Th. During further qualitative analysis, data from these three measurements were merged into one data file so that each analytical group was characterized by a single set of proteins. Statistical analysis concerning mass spectrometry was performed as described in the protein quantification section and described in more detail previously [40].

### Protein identification

The acquired MS/MS data were pre-processed with Mascot Distiller software (v. 2.5, Matrix Science), and a database search was performed using the Mascot Search Engine (Matrix Science, Mascot Server 2.4.1) against the SwissProt database (release 2015_01, 547,357 sequences; 194,874,700 residues) restricted to human proteins (20,274 sequences). To reduce mass errors, the peptide and fragment mass tolerance settings were established separately for individual LC–MS/MS runs after a measured mass recalibration. Other Mascot search settings were as follows: enzyme—semiTrypsin, missed cleavages—1; fixed modifications: Methylthio (C); and variable modifications: oxidation (M). A statistical assessment of peptide assignments was based on the concatenated target/decoy database search strategy (merged target/decoy databases generated with software developed in-house [40]). This procedure (Supplementary Method 2) provided q-value estimates for each peptide spectrum match (PSM) in the dataset. All PSMs with q-values > 0.01 were removed from further analysis. A protein was regarded as confidently identified when at least two peptides of this protein were found. Proteins identified by a subset of peptides from another protein were excluded from analysis. Proteins that exactly matched the same set of peptides were combined into a single group (cluster). The mass calibration and data filtering described above were carried out with Mscan [41] software, developed in-house. Peptide/protein identifications derived from all analytical groups were exported and used in further quantitative analysis.

### Protein quantification

Label-free quantification was performed essentially as described in [40] using 2D heat-maps generated from LC–MS profile datasets and the list of protein/peptide identification. The abundance of each peptide was determined as the height of a 2D fit to the monoisotopic peak of the isotopic envelope. Quantitative values were next exported into text files, along with peptide/protein identifications into pairwise statistical analysis using Diffprot software [40]. A non-parametric resampling-based test statistics with local variance estimate makes Diffprot an appropriate tool for analysis of small scale biological experiments. Diffprot was run with the following parameters: number of random peptide sets = 106; clustering of peptide sets with 80% similarity or higher; and normalization by LOWESS. Results for proteins present in one of the analyzed groups in the pairwise comparison were manually validated on the heat-maps.

The mass spectrometry proteomics data were deposited to the ProteomeXchange Consortium [42] via the PRIDE partner repository using the dataset identifiers XD002600 and 10.6019/pxd002600*.

### Electrophoresis and western blotting

HKCSCs cells cultured in T25 flasks as described in “Suspension cell cultures”. For clear-cell RCC positive control, 769-P (primary ccRCC) and Caki-1 (ccRCC metastatic) cells were cultured in T75 flasks in monolayer. Cells were collected, washed once in PBS and lysed in 50 µl of RIPA buffer. Undissolved proteins were pelleted and protein concentration in lysates was measured with BCA assay (Sigma Aldrich). Ten µg of total proteins from samples was dissolved after extraction in 4× Laemmli Sample Buffer (60 mM Tris–Cl pH 6.8, 2% SDS, 10% glycerol, 5% β-mercaptoethanol, 0.01% bromophenol blue, 1:3, Amresco Cat. No. J60015). Electrophoresis was performed in polyacrylamide TruPAGE™ Precast 12% gels in SDS-PAGE buffer. Subsequently, gels were transferred onto Nitrocellulose membranes (biorad, Cat. No. 162-0094); protein transfer and loading was confirmed by Ponceau S staining (Sigma, Cat. No. 09189). After washing, membrane was blocked with Nonfat Powdered Milk (Amresco, Cat. No. M203) and then probed with primary antibodies: anti-eif4b (D-4) (Santa-Cruz Biotechnology, Inc., Cat. No. sc-376062), anti-map2k1 (Sigma-Aldrich, Cat. No. HPA026430). Detection was performed after incubation with secondary antibodies: goat anti-rabbit IgG–HRP (Cell Signalling Technology, Cat. No. 7074) or goat anti-mouse IgG–HRP (Santa Cruz, Cat. No. sc-7023), according to recommendations from the manufacturer using chemiluminescent horseradish peroxydase substrate Luminol (SantaCruz Cat. No. sc-2018) using X-ray films. Prism Ultra Protein Ladder (10–245 kDa) (Abcam, Cat. No. ab116027) was used for protein size estimation. Membranes were scanned and analyzed with ImageJ. Band densities were related to Ponceau S staining (reference band: 70 kDa).

### Real-time PCR

Cells were cultured in T25 cell culture flasks in monolayers; after 72 h incubation, cells with approximately 80% confluence were washed with PBS and covered with Fenozol reagent (Total RNA Mini Plus kit, A&A Biotechnology, Gdynia, Poland) and RNA isolation was performed according to the manufacturer’s protocol. Genomic DNA was removed with TURBO DNA-free Kit (Ambion). The concentration and purity of RNA were determined by measuring the absorption at 230, 260, and 280 nm in a Multiskan™ GO Microplate Spectrophotometer. SuperScript IV First-Strand Synthesis System (Invitrogen) was used to generate full-length first strand cDNA from total RNA templates using a mixture of oligo-(dT) primers and hexamers. 2.5 μg of total RNA in 20 μL of reaction mix was used for reverse transcription. Single strand complementary DNA (cDNA) generated in rtPCR was used as a template for amplification by sqPCR. Real-time PCR was performed either with PowerUp SYBR Green Master Mix or TaqMan Gene Expression Master Mix (both Applied Biosystems) in 7500 Real-Time PCR System (Applied Biosystems) in 96 well plates. Tested genes included: *flt1* (*vegf*-*1*, forward CCTGCAGAGCCAGGAATGTAT, reverse GTTGCAGTGCTCACCTCTGA), *b*-*raf* (forward AAAACACCCATCCAGGCAGG, reverse ACTCTCCTGAACTCTCTCACTCA) and *c*-*kit* (Hs00174029_m1 TaqMan probes). For the confirmation of no contamination with gDNA, minus-RT controls were performed for each sample. Data were calculated with the 2(−Delta C(T)) method, with normalization to mean expression of peptidylprolyl isomerase A (PPIA) as a housekeeping gene (sequences for SYBRGreen assay: forward CGTGGTATAAAAGGGGCGG, reverse, TGTCTGCAAACAGCTCAAAGG, assay Hs01565699_g1 for TaqMan probes).

### Statistical analysis

For Alamar blue statistical measurements, ANOVA test was used (all results from all 4 doses from day 1 were compared with all results from all 4 doses from day 6 of the experiment). Statistically significant results are marked with * when P < 0.001. For mass spectrometry protein quantification, Diffprot software [40] was used as described in “Protein quantification” section. For Muse™ flow cytometry analysis, ANOVA test was used (statistically significant results are marked with * when P < 0.001 and ** when P < 0.05). Error bars on all figures are presented as mean ± SD.

## Results

### Specific oxygen concentration induces different TKI response

**Fig. 2:**
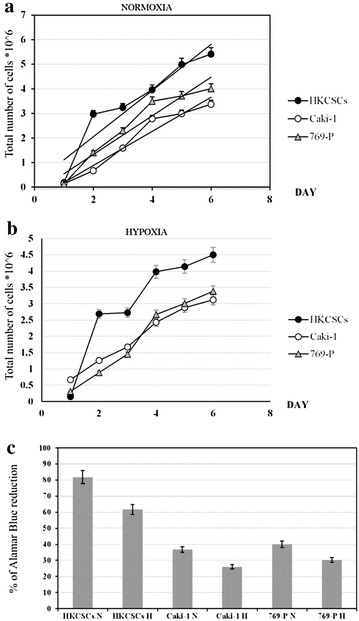
HKCSCs papillary stem-like cell line has higher proliferation rate than 769-P and ACHN clear-cell RCC cell lines both in normoxia (**a**) and hypoxia (**b**) culture conditions. Living cells were counted using Bürker chamber during 6 days. Those results are confirmed by % of Alamar blue® reduction in HKCSCs, 769-P and Caki-1 untreated cell lines in normoxia vs hypoxia (**c**). Error bars presented as ± SD

**Fig. 3:**
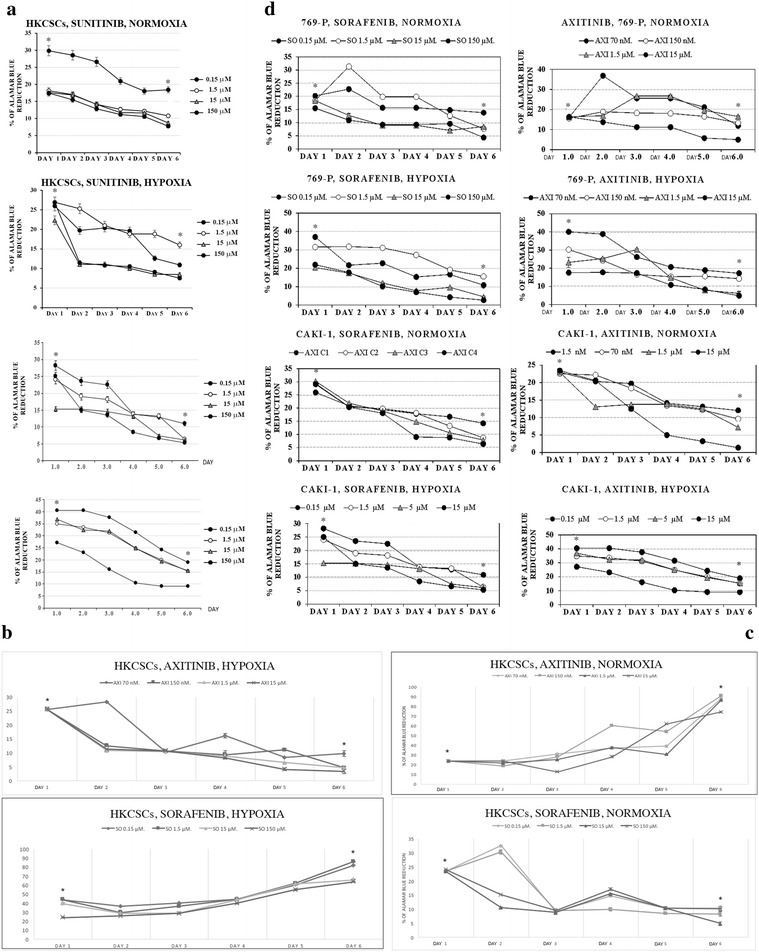
Cell viability by Alamar blue® of HKCSCs, 769-P and Caki-1 cells treated with various doses of sunitinib (**a**) reduces overtime. Only HKCSCs are resistant to sorafenib in hypoxia, marked *rSH* (**b**) and to axitinib in normoxia, marked *rAN* (**c**) while 769-P and Caki-1 remain sensitive to treatment as indicated for 6 days (**d**). Graphs show the % of Alamar blue® reduction which indicates the number of living (metabolically active) cells. Statistically significant results are marked with * when P < 0.001 (ANOVA test). Two graphs marked with a square show resistant cell lines. Error bars presented as ± SD

769-P and Caki-1 cells showed reduced proliferation rates under treatment with sunitinib, sorafenib and axitinib in monolayer cell cultures both in normoxia and in hypoxia (Figs. 2, 3a–d). Proliferation inhibition is dose-dependent, with a significantly ccRCC-toxic minimal dose of 1.5 nM axitinib for the Caki-1 cell line and a maximal dose of 150 µM sorafenib for the 769-P cell line (both in normoxia). Also, papillary renal cancer cells—HKCSCs were directly and effectively inhibited with sunitinib in monolayer cell cultures. Sunitinib-mediated inhibition of HKCSCs proliferation was shown to be dose-dependent with IC_50_ ~ 10.5 µM in normoxia and ~ 0.75 µM in hypoxia (Fig. 3a–d).

Proliferation inhibition was also shown to be dose-dependent within ccRCC cell lines, with IC_50_ ranging from ~ 0.20 to ~ 13.2 µM for 769-P cells cultured in sunitinib and for Caki-1 cells cultured in sorafenib, respectively, both in normoxic conditions. However, it should be noted that axitinib is more toxic than sorafenib and sunitinib; therefore, lower doses are needed to obtain the same proliferation reduction rate (around 10× lower in the case of axitinib than sorafenib or sunitinib). Thus, activity of axitinib against RCC cell lines is higher than for sunitinib/sorafenib, reaching IC_50_ at the concentration of minimum 70 nM (769-P in hypoxia) and maximum of ~ 0.13 µM (Caki-1 in hypoxia). IC_50_ was counted for day 6 of the 2D cell culture. All results reached statistical significance (P < 0.001).

We have observed that papillary RCC HKCSCs cells possibly developed primary-like resistance phenomenon to sorafenib due to hypoxic conditions (*rSH*) and to axitinib in standard oxygen concentration (*rAN*). HKCSCs were not resistant to axitinib in hypoxia and thus remained sensitive (*sAH*). HKCSCs exhibited the same relationship with sorafenib in normoxia (*sSN*). Sorafenib and axitinib effectively inhibited the growth of primary and metastatic clear-cell renal cell carcinoma cell lines in normoxia and hypoxia, but pRCC stem-like cells’ growth was inhibited in an oxygen-dependent manner (Fig. 3d).

**Fig. 4:**
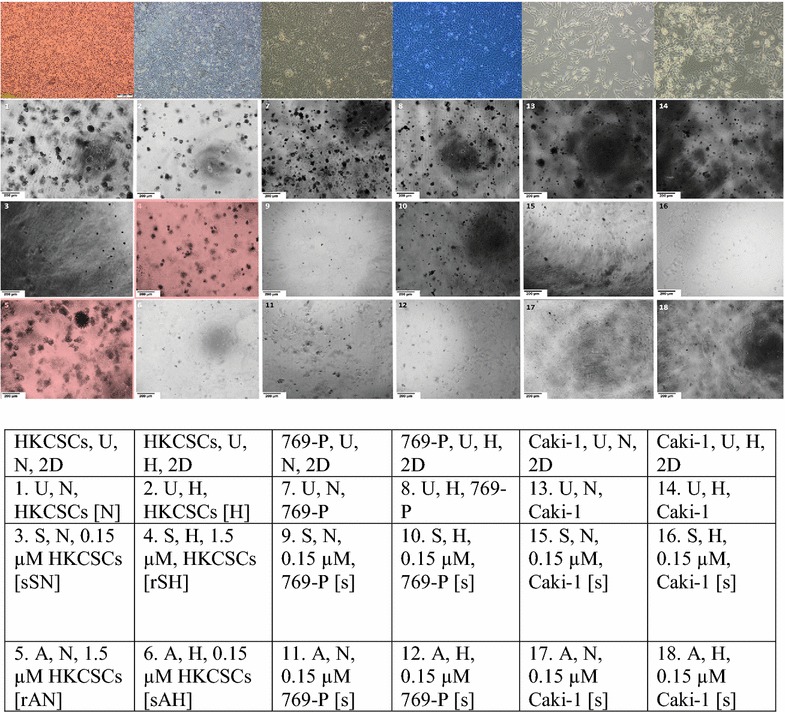
HKCSCs cells are resistant to sorafenib in hypoxia and to axitinib in normoxia. Hypoxia decreases the amount of colonies visible in 96-well plates. Representative images of colonies (performed in triplicate). Magnification 4×, scale bar = 200 µm. The scheme of the figure is shown below. Above 2D monolayer cell culture phenotypic view is presented for comparison. *U* untreated cells, *N* normoxia, *H* hypoxia, *r* resistant cells, *s* sensitized cells, *A* axitinib, *S* sorafenib. Resistant HKCSCs are marked with a red square

Stem cell colony formation assay was performed only with sorafenib and axitinib to confirm the impact of oxygen concentration on renal cancer cells’ response to tyrosine kinase inhibitors. Regardless of oxygen concentration, HKCSCs, 769-P and Caki-1 formed colonies if no TKI treatment was applied, however, hypoxic colonies were in general smaller and did not develop necrosis. Cells inside the colonies were living cells based on Neutral Red uptake (data not shown). Colonies formed by Caki-1 were of larger volume but in smaller quantity than colonies formed by 769-P cells. The highest number and largest size of colonies were observed in the HKCSC culture. 769-P and Caki-1 cell lines cultured with sorafenib, and axitinib had a reduced size of colonies and underwent cell death, whereas resistant HKCSCs colonies were still increasing in size and number on day 30, when the cell culture was terminated as innumerable live colonies were formed (based on Neutral Red uptake, data not shown) (Fig. 4).

Hypoxia slightly decreased the size and amount of colonies that were not subjected to TKIs and significantly decreased the size and amount of axitinib but not sorafenib-treated 769-P colonies. Interestingly, Caki-1 colonies behaved exactly the opposite; under hypoxic conditions, the size and amount of sorafenib, but not axitinib-treated colonies, were visibly decreased.

**Fig. 5:**
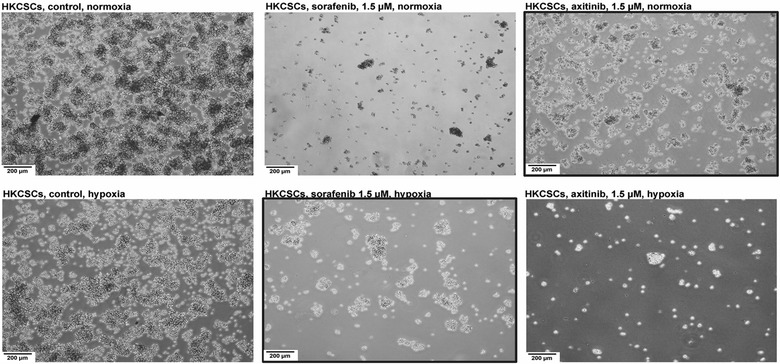
Heterogenous aggregate formation in suspension culture of HKCSCs before protein isolation for mass spectrometry and Western blotting at day 6 of the culture prove resistance of HKCSCs to sorafenib in hypoxia and axitinib in normoxia. Reduced proliferation rates in lower oxygen tension is shown. Resistant HKCSCs are marked with a black square. Magnification: 4×, scale bar = 200 µm

In suspension culture (the second 3D model) in which only HKCSCs were used to investigate the effect of hypoxia on TKI-treated RCC cells in another 3D in vitro model, multiple aggregates both before and after TKI treatment were formed. Hypoxia slightly decreased the size and amount of cell aggregates–interestingly, three-dimensional structures were darker in normoxia, which suggested necrosis but it was not confirmed by Neutral red uptake (data not shown). The size of aggregates did not change or decreased slowly in TKI resistant cells (*rAN, rSH*), while in untreated cells the number of aggregates was stably increasing and in sensitized cells (*sSN, sAH*) a reduction in the number of aggregates was observed (Fig. 5).

### Oxygen tension regulates renal cell carcinoma proliferation and viability

**Fig. 6:**
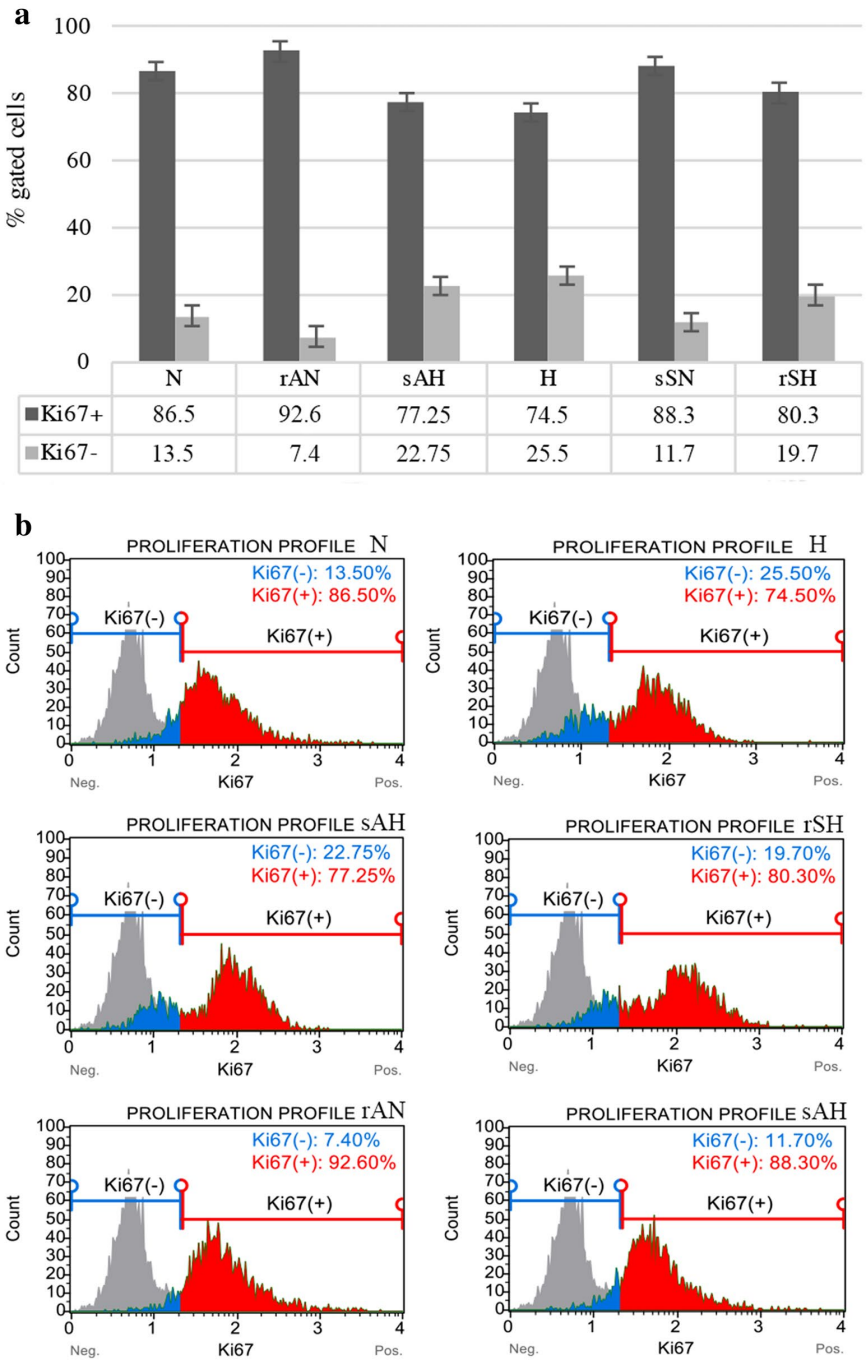
Reduced number of proliferating HKCSCs cells in hypoxia compared to normoxia according to flow cytometry histograms gated on Ki67+ cells (**a**). Bar plots are presented (**b**). Error bars presented as mean ± SD

Hypoxia was shown to reduce RCC cells’ proliferation rate and viability in 2D and 3D cell culture models. HKCSCs, 769-P and Caki-1 cells cultured in monolayer in hypoxia, irrespectively of TKI treatment, showed a reduced rate of proliferation in comparison to cells cultured in normoxic conditions as shown by resazurine-based viability test (Fig. 3b–d). This effect was confirmed with enumeration of live HKCSCs, 769-P and Caki-1 cells in the 2D culture where all cells reached higher number in normoxia (Fig. 2a) than in hypoxia (Fig. 2b). Proliferation rate was also defined by Ki67 expression in suspension HKCSCs cultures. HKCSCs cultured in normoxia were Ki67 positive (Ki67+) in 87% in comparison with HKCSCs cultured in hypoxia—74%, irrespective of TKI treatment (Fig. 6a, b). HKCSCs had the highest proliferation rate compared with 769-P and Caki-1 cell lines; clear-cell renal cancer cells had a proliferation rate of about 28% lower than HKCSCs, in both oxygen levels (Fig. 6a, b).

### Tyrosine kinase inhibitors resistance–related response in renal cancer stem cells

**Fig. 7:**
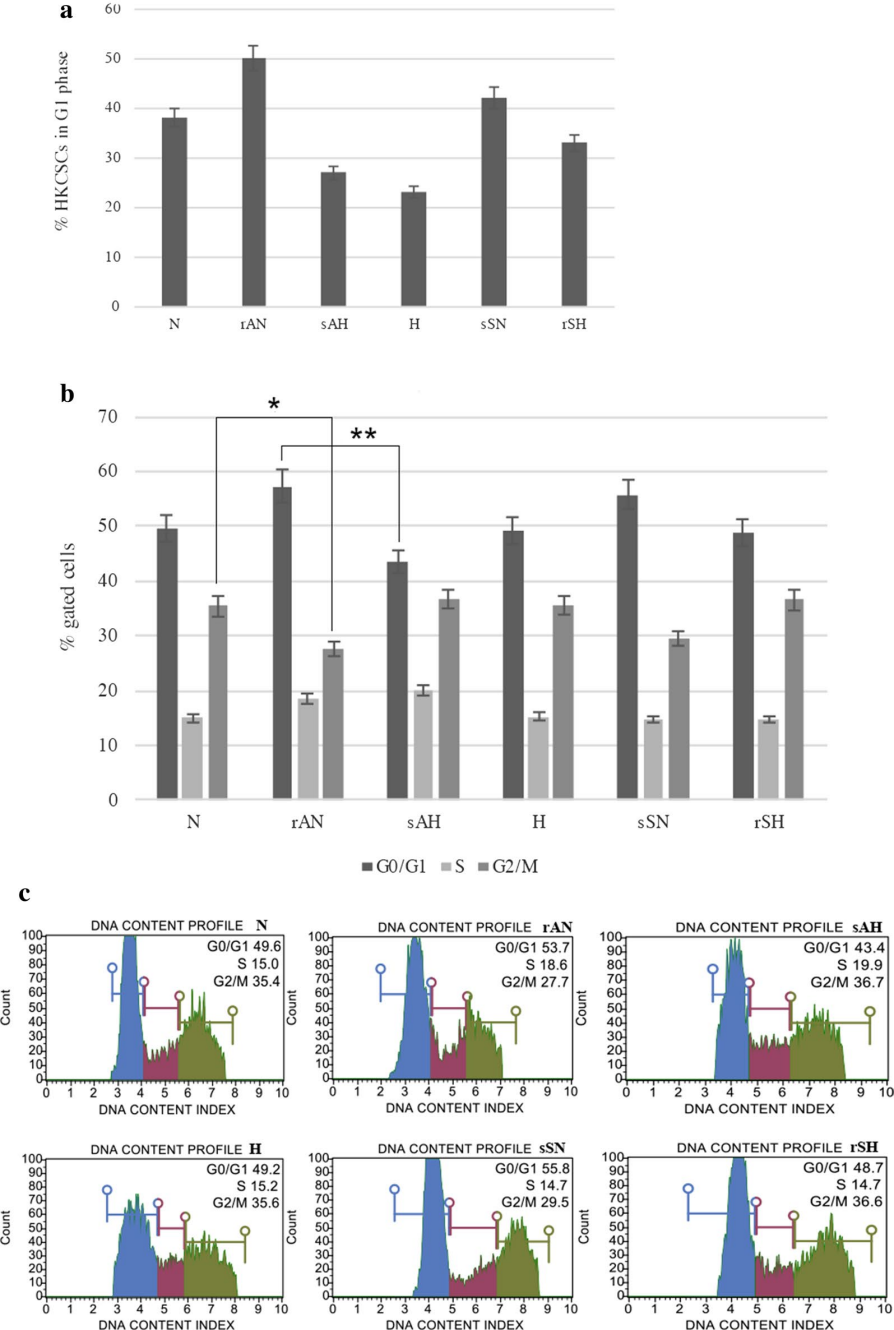
% of G_1_ HKCSCs cells counted on the basis of the equation: G_1_ = (G_0_/G_1_ − G_0_:Ki67−) show G_1_ HKCSCs cells (**a**) and percentage of HKCSCs cells, resistant, sensitized and untreated with sorafenib and axitinib entering each of cell cycle phases (G_0_/G_1_, S. G_2_/M) (**b**). Bar plots are presented (**c**). Error bars presented as mean ± SD. Statistically significant results are marked with * when P < 0.001 and ** when P < 0.05 (ANOVA test)

Reduced proliferation of HKCSCs cells in hypoxic conditions was observed alongside an increase in the number of quiescent (Ki67−) cells and cells arrested in the S phase. In sorafenib-resistant cells *(rSH)* in hypoxia, the percentage of Ki67− cells was lower than in untreated hypoxic cells *(H).* In axitinib-resistant cells in normoxia, more cells expressed Ki67 and entered G1 phase in comparison to non-treated cells (*rAN* vs *N*). Sorafenib-sensitive cells (*rSH*) in normoxia displayed S phase arrest upon treatment. Differences in HKCSCs cell cycling were found between normoxic and hypoxic cells, irrespectively of TKI treatment. In the case of sorafenib, hypoxic conditions promoted cell survival as opposed to axitinib treatment for which low oxygen pressure sensitized cells to treatment which was shown in 2D cell cultures (Fig. 3d) as well as in soft agar suspension culture, where red squares show resistant cells (see Fig. 4). Also, percentage of cells in G1 phase is the highest in resistant cells—rAN (Fig. 7a), as well as significantly higher percentage of G0/G1 cells was noticed for resistant than for sensitized cells (Fig. 7b, c).

### Elevated map2k1 expression in hypoxia compared to normoxia in TKI treated HKCSCs cells

**Fig. 8:**
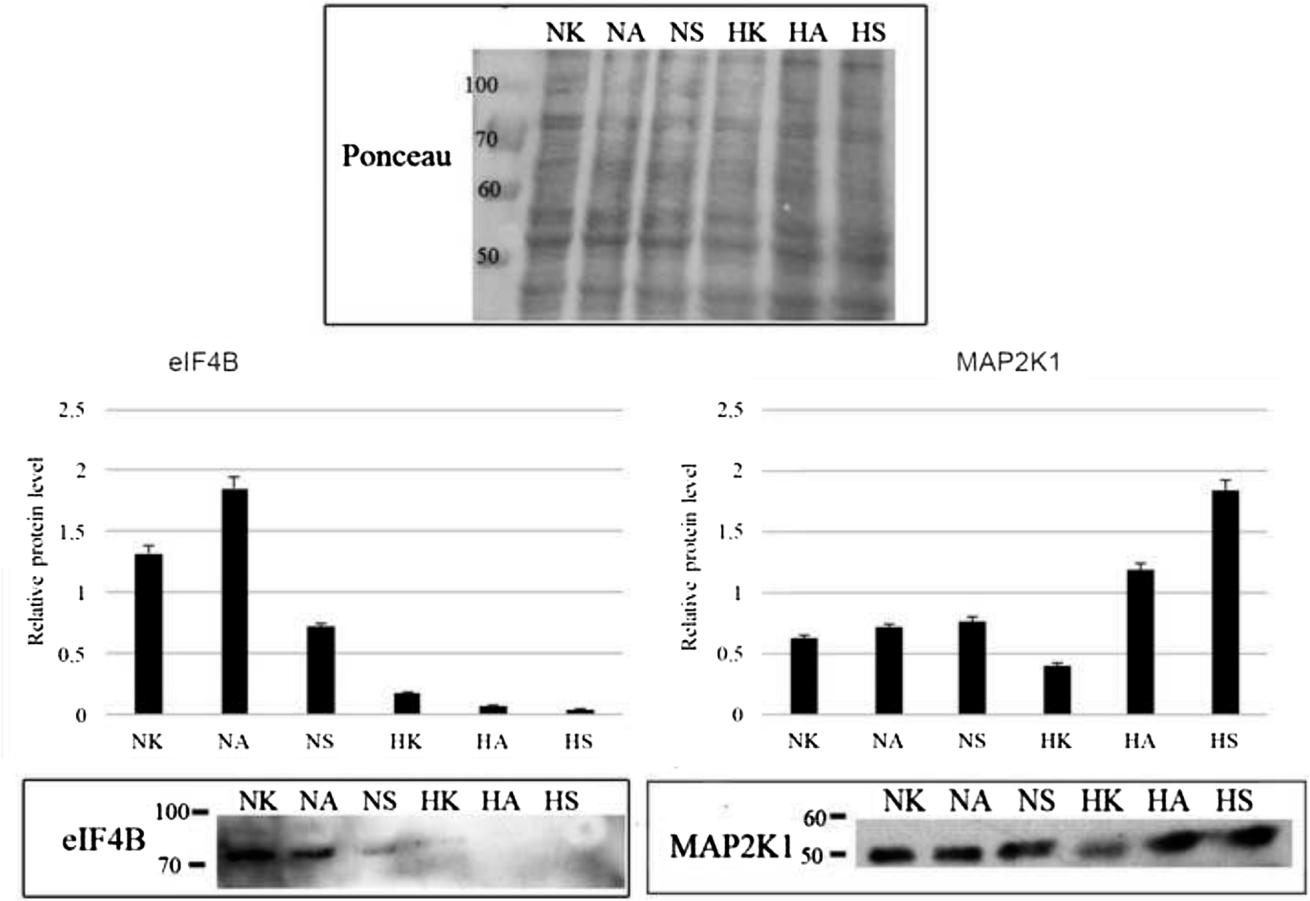
Bar graphs represent relative band intensity of map2k1 and eif4b. Quantified mean protein value is relative to Ponceau which was used for normalization. To account for the difference in protein loading during the experiment, the percentage of regulation was calculated after the intensity of each band was adjusted according to its respective Ponceau band intensity using the ImageJ 2.1.4.7. software (National Institutes of Health). Representative western blots are depicted, showing map2k1 and eif4b expression in HKCSCs untreated, resistant and sensitized cells in normoxia and hypoxia. Error bars presented as mean ± SD

Dual specificity mitogen-activated protein kinase 1 (encoded by *map2k1* gene) protein was detected on high level in HKCSCs, characterized by a different reaction to TKIs depending on pO2. In 769-P the protein expression was much lower, while in Caki-1 cells the protein could not be detected (Fig. 8). Additionally, elevated expression of map2k1 was observed in HKCSCs treated with tested drugs in hypoxia, while in normoxia sorafenib and axitinib weakly altered the level of the protein. This protein was previously reported in RCC [43, 44]. This finding corresponds to the fact that *map2k1* is a *mek*-*1* kinase targeted by sorafenib; until now it was known that sorafenib does not inhibit *mek*-*1* kinase activity in vitro [13]. Map2k1 was detected only in hypoxic sample in mass spec analysis (Table 1).

**Table 1:** Mass spectrometry analysis results of chosen proteins

Protein name	Gene name	Full name	qvalue	Ratio	Fold change	Peptide no.	Citation (protein association with RCC or cancerogenesis or hypoxia)	Function (main process)
P23588	***eif4b***	Eukaryotic translation initiation factor B	NA	SH/AN: only rSH N: 0 rAN: 0 H: 0 rSH: 1	NA	2	[57]	Signal transduction
Q02750	***map2k1***	Dual specificity mitogen-activated protein kinase 1	NA	N/H: only H N: 0 H: 1 rSH: 0 rAN: 0	NA	5	[58, 59]	Signal transduction
Q99459	*cdc5* *l*	Cell division cycle 5-like protein	NA	Only rSH (rSH/rAN)	NA	2	[60]	Cell cycling
O60264	*smarca5*	SWI/SNF-related matrix-associated actin-dependent regulator of chromatin subfamily A member 5	NA	Only rSH (rSH/rAN)	NA	3	[61]	Nucleosome-remodeling activity
Q9UIG0	*baz1b*	Tyrosine-protein kinase BAZ1B	NA	Only rAN (AN/N)	NNA	2	[62]	Chromatin remodeling
P07910	*hnrnp*	Heterogene-ous nuclear ribonucleo-proteins C1/C2	NA	Only rAN (rAN/N)	NNA	2	[63]	Regulation of mRNA splicing
P14618	*pkm*	Pyruvate kinase PKM	0.00013 (H/N) 0.00011 (rSH/rAN)	2.34 (H/N) 1.89 (rSH/rAN)	2.34 (H/N) 1.89 (rSH/rAN)	45 (H/N) 42 (rSH/rAN)	[64]	Metabolic (production of ATP)
P05091	*aldh2*	Aldehyde dehydroge-nase, mitochon-drial	NA	Only H (H/N) Only rAN (rAN/N)	NA	4 (H/N) 3 (rAN/N)	[65]	Alcohol metabolism, marker for stem cells
Q9BXP5	*srrt*	Serrate RNA effector molecule homolog	NA	Only H (SH/H)	NA	2	[66]	Cell proliferation
P62258	*14*-*3*-*3ε*	14-3-3 epsilon	0.00027	1,7 (rSH/rAN)	1,7 (rSH/rAN)	21	[67]	Is believed to play a role in G2/M transition
Q9NYF8	*bclaf1*	Bcl-2-associated transcription factor 1	NA	Only rSH (rSH/H)	NA	2	[68]	Apoptosis, negative regulation of transciption
P00338	*ldha*	L-lactate dehydrogenase A chain	0.00011 (SH/AN) 0.00816 (H/N)	2.54 (rSH/rAN) 1.91 (H/N)	2.52 (rSH/rAN) 1.91 (H/N)	17 (rSH/rAN) 16 (H/N)	[69]	Metabolic—higher levels in cancer

Those marked in bolditalic were chosen for western blotting

P* is not counted when protein is detected in only one group (100%). qvalue represents the statistical significance. The lower qvalue, the higher statistical significance. NA (not applicable) means that the protein was detected only in one group in a particular group comparison. If the protein was not a 0–1 protein, protein ratio is given. For example, there is 2.34 more amount of P14618 protein encoded by *PKM* gene in the hypoxia group but only when we compare it to the amount in the normoxia group

*rAN*: cells resistant to axitinib in normoxia, *sAH*: cells sensitized to axitinib in hypoxia, *N*: cells cultured in normoxia, *H*: cells cultured in hypoxia, *rSH*: cells resistant to sorafenib in hypoxia, *sSN*: cells sensitized to sorafenib in normoxia

### Reduced eif4b expression in hypoxic HKCSCs cells

Also eif4b had the strongest expression in HKCSCs. In normoxic cells resistant to axitinib the level of protein was upregulated, while sorafenib decreased it. In all tested hypoxic samples the expression of eif4b was strongly diminished (Fig. 8). In total, hypoxic effect is similar in both proteins—while it decreases the expression of eif4b protein regardless of TKI treatment, and decreases the expression of map2k1, in the case of map2k1 both TKIs were able not only to restore it, but also to even elevate it.

### Expression of TKI targets in RCC cells

**Fig. 9:**
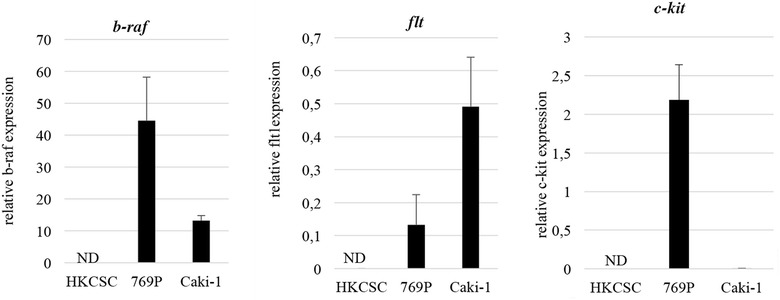
Real-time PCR analysis of TKI receptor targets. Error bars presented as mean ± SD

Tyrosine kinase inhibitors therapeutical effect in solid tumors is primary mediated by the action of the drugs on endothelial cells as anti-angiogenic therapy. However, direct effects of these drugs on cancer cells have been reported previously [20, 35]. Yet, the mechanism of this phenomenon is not clear. Therefore, we checked whether studied RCC cell lines express some of the genes targeted by tested TKIs. Since *braf and c*-*kit* are targeted by sorafenib but not by axitinib, and *vegfr*-*1* is targeted by axitinib but not by sorafenib, those were receptors of choice when assessing the receptor repertoire expression. No detectable expression of each tested genes was observed in HKCSCs on the level of transcript. In our preliminary research, we have detected several surface receptor markers expression in HKCSCs using Real-time PCR method, i.e. *cd133* and *cd105* under sorafenib treatment and without treatment in normoxia and hypoxia (data not shown). 769-P cells showed higher level of *b*-*raf* and *c*-*kit* than Caki-1 cell line, while *flt1* (also called *vegfr*-*1* gene) expression dominated in metastatic cell line (Fig. 9).

## Discussion

Current availability of several effective agents allowed an extended OS (overall survival) rate in RCC patients, but mostly in patients with clear-cell RCC [16]. In our study, in 2D models, hypoxia does not alter cell morphology, but in the 3D level hypoxia reduces 3D structures’ amount and volume leaving the cells inside the 3D structure alive. Since the cells’ lysosomes incorporated Neutral Red stain, alive cells are present inside cell aggregates [45]. In such oxygen-restricted conditions, papillary renal cell carcinoma cells develop primary-like resistance to sorafenib and alter its cell cycling and expression of crucial proteins, which have been shown in this study. Moreover, hypoxia causes papillary RCC cells (HKCSCs) to behave differently under sorafenib and axitinib treatment in three independent cell culture models which may suggest that those cells adapt to the restricted niche and are predetermined to form a tumor.

Herein, altered protein expression was mainly observed in cells cultured in lower oxygen tension regardless of TKI treatment and regardless of whether papillary renal cancer cells developed resistance (Table 1). Therefore, it seems that the main factor contributing to protein in vitro expression switch is low oxygen tension rather than drug treatment. On the other hand, in papillary RCC resistant to sorafenib in hypoxia, map2k1 expression was highly elevated when compared to untreated and sensitized samples. We have yielded similar results in four biological repeats (data not shown). Nonetheless, our main finding was that although hypoxia decreased the expression of map2k1, a *mek1* kinase, the treatment with axitinib and sorafenib has on the contrary increased map2k1 expression. Resistant cells to sorafenib in hypoxia had the highest map2k1 expression level. Such phenomenon was already presented in the literature [46]. Overall, it seems that both resistant and sensitized phenotypes of HKCSCs cells up-regulated this protein.

Since map2k1 is responsible for signal transduction, it possibly directed cells observed in G1 phase to synthesize and divide—subsequently, the cells halted the proliferation in S phase arrest. It may suggest that this mechanism may be responsible for resistant cells’ unique way of survival, which does not take place in normoxia and in this way further studies regarding hypoxia should be implemented while studying drug resistance phenomenon.

Previously described alterations in map2k1 protein expression together with eif4b support the hypothesis that key proteins expression in hypoxic tumor niche is regulated mostly by oxygen tension. These findings support the crucial role of mek1 kinase may be useful in cancer cells response to antiangiogenic agents. It was previously reported that inhibition of mek1 in the renal cell carcinoma xenograft model with acquired resistance to sunitinib successfully improved anti-tumor drug efficacy [47]. Therefore, targeting mek1 may be a promising pathway in treating TKI-resistant papillary renal cancer patients.

Our results confirm previously published clinical data, which showed eifs expression in advanced cancers *inter alia* in renal cell carcinomas [48]. The role of eif4b has not been thoroughly investigated until now, nor has the effect of tumor hypoxia on its expression and vice versa. In our study, overexpression of eif4b was observed upon treatment both in axitinib resistant (rAN) and cells sensitized to sorafenib (sSN) in normoxia. Hypoxic conditions down-regulated the protein, in consequence attaining phenotype sensitive to axitinib. However, hypoxic cells retained drug resistance to sorafenib. The activity of eifs was previously reported to promote survival of cancer cells [49]. Interestingly, the addition of axitinib in resistant normoxic cells elevated eif4b expression, which would support the hypothesis that hypoxic conditions abrogate eif4b expression regardless of TKI addition.

As a result of hypoxia-response mechanism, metabolism of the cell switches to aerobic glycolysis parallely with high glucose influx and high production of lactate, which occurs in cancer cells [50]. Until now, it is known that 24% of proteins altered in hypoxia are metabolic [51]. Our study showed that pkm, lhda and aldh2, which are all metabolic proteins, were overexpressed in hypoxia–cultured papillary HKCSCs regardless of TKI treatment, being at the same time underexpressed in normoxic groups: *rAN*, *N* and *sSN*. This finding supports current results published in the literature [52]. Among differentially expressed proteins with statistically significant results (P < 0.001* or the protein was identified in only one group), we detected those implicated in cell cycling and proliferation (cdc5l, srrt, 14-3-3ε), transcription and/or translation (bclaf1, hnrnpc) and chromosome remodeling (baz1b, smarca5) (Table 1).

What is more, none of the tested TKI targets were expressed in HKCSCs so it may be that direct anti-cancer effects of sorafenib and axitinib in studied conditions is mediated by other receptors. It was reported that most TKIs are active also against other, off-target tyrosine kinase signalling pathways [53]. In total, the identification of hypoxia-dependent RNA–protein complexes such as eif4b and map2k1 and their molecular characterization in cancer signaling should provide insight into new pathways that may modulate translation rates in resistant and hypoxic cancer cells [54].

Not only is papillary renal cancer cells’ protein expression profile affected by hypoxic conditions, but 1% pO_2_ also impacts papillary RCC cells’ proliferation rate, cell cycling and response to tyrosine kinase inhibitors, which we confirmed in both the 2D and 3D cell cultures. Papillary RCC stem-like cells respond differently to axitinib and sorafenib. These cells are resistant to axitinib in normoxia *(rAN)* and resistant to sorafenib in hypoxia *(rSH).* This is, however, primary resistance, not induced by exposing cells to gradually increasing TKI doses, as presented in a previous in vitro study on sunitinib in the 786-O cell line [55].

Hypoxia increases the number of Ki67− cells and cells in S phase and decreases the number of cells in G_1_ phase. We suggest that as a result of hypoxic conditions, cells divide at a slower rate and reach a quiescent state (G_0_ phase [61]) or arrest in S phase. We reported an increased percentage of only resistant cells (*rAN* and *rSH*) entering G_1_ cell cycle phase and a simultaneous drop of G_0_ cells, which may suggest that quiescent cells are reentering cell cycle as a response to the drugs [56]. At the same time, these cells are arrested in the S phase, with no change in G_2_/M fraction. Interestingly, in sensitized cells (*sSN*, *sAH*) TKI treatment does not affect the cell cycle.

Slower proliferation rate of hypoxic cells presented in this research causes higher cells’ resistance to apoptosis and/or necrosis. Cells growth is slower; therefore, cell aggregates do not reach the sized of normoxic aggregates, allowing hypoxia to reach similar level in each of 3D structure site. This slower growth may allow resistant cells to adapt even to very rigorous conditions and to high drug concentrations, which seems to have much in common with the general tumorigenic process.

## Conclusions

In conclusion, hypoxia impacts tyrosine kinase inhibitors—treated clear-cell and non- clear-cell renal cancer cells, papillary and clear-cell. Stem-like papillary RCC cells gain primary-like resistance phenotypic features dependent on oxygen concentration as well. Thus, oxygen tension regulates tyrosine kinase as well as RCC cells’ proliferation rate, protein and gene expression profile. These findings suggest that hypoxia modulates renal cancer types’ treatment efficacy, also other than clear-cell, which may be primarily investigated in vitro. Nonetheless, there is a need to focus on many potential molecular targets both on the in vitro and in vivo level, since protein activity in vitro may not recapitulate in vivo conditions, as in the map2k1 case. We can also hypothetise that map2k1 may play a role in hypoxia-mediated resistance to sorafenib, while eif4b expression may have its role in resistance to axitinib as observed in normoxia.

Nonetheless, protein expression profile needs to be double-checked in laboratory conditions and in clinical trials. To avoid opposite results in vitro and in vivo, novatory hypoxic in vitro models need to be established.
